# The Utility of the Health Action Process Approach Model for Predicting Physical Activity Intentions and Behavior in Schizophrenia

**DOI:** 10.3389/fpsyt.2017.00135

**Published:** 2017-08-04

**Authors:** Kelly P. Arbour-Nicitopoulos, Markus J. Duncan, Gary Remington, John Cairney, Guy E. Faulkner

**Affiliations:** ^1^Faculty of Kinesiology and Physical Education, University of Toronto, Toronto, ON, Canada; ^2^School of Kinesiology, University of British Columbia, Vancouver, BC, Canada; ^3^Schizophrenia Program, Centre for Addiction and Mental Health, Toronto, ON, Canada

**Keywords:** schizophrenia, physical activity, determinants, theory based, accelerometry

## Abstract

Research is needed to develop evidence-based behavioral interventions for preventing and treating obesity that are specific to the schizophrenia population. This study is the precursor to such intervention research where we examined the utility of the social cognitions outlined within the Health Action Process Approach (HAPA) model for predicting moderate to vigorous physical activity (MVPA) intentions and behavior among individuals with schizophrenia or schizoaffective disorder. A prospective cohort design [baseline (T1), week 2 (T2), and week 4 (T3)] was used to examine the HAPA constructs and MVPA across a sample of 101 adults (*M*_age_ = 41.5 ± 11.7 years; *M*_BMI_ = 31.2 ± 7.8 kg/m^2^; 59% male). Two hierarchical regression analyses were conducted controlling for age, gender, BMI, and previous self-reported MVPA. In the first regression, intentions at T1 were regressed onto the T1 motivational HAPA constructs (risk perception, affective attitudes, task self-efficacy) and social support; MVPA status (meeting vs. not meeting the MVPA guidelines) assessed via accelerometry at T3 was regressed onto T1 social support and intentions followed by T2 action and coping planning, and maintenance self-efficacy in the second analysis. Overall, the motivational and social support variables accounted for 28% of the variance in intentions, with affective attitudes (β = 0.33, *p* < 0.01) and task self-efficacy (β = 0.25, *p* < 0.05) exhibiting significant, positive relationships. For MVPA status, the model as a whole explained 39% of the variance, with the volitional HAPA constructs explaining a non-significant 3% of this total variance. These findings suggest a need for interventions targeting self-efficacy and affective attitudes within this clinical population.

## Introduction

Recent data indicate 13–15 years of life lost to schizophrenia compared to the general population (1). Cardiovascular death is a major contributor to this increased mortality (2). Potential causes of this excess mortality are varied, although they can be broadly categorized in terms of the iatrogenic effects of treatment (e.g., metabolic side effects of medication), greater prevalence of engagement in unhealthy behaviors (e.g., smoking, physical inactivity), and limited access to health care (3). Given the compelling evidence that physical activity prevents premature mortality, cardiovascular disease, and type 2 diabetes in the general population (4), one increasing focus of attention has been attempts to reduce physical inactivity in this population. Despite the many potential physical, psychosocial, and cognitive benefits of physical activity for individuals with schizophrenia (5), individuals with schizophrenia engage in significantly less moderate and vigorous physical activity compared to controls (6). Research is now required to inform the development of evidence-based behavioral interventions for increasing physical activity that are tailored to this population (7).

Theory-based physical activity interventions in the general adult population have been shown to be more effective at increasing physical activity than atheoretical interventions (8). A theoretical framework provides guidance in how to develop and implement interventions, identifies key modifiable constructs to target in intervention work, and informs measurement development to confidently assess how well interventions are influencing potential constructs mediating behavior change. Researchers have begun to examine theoretical factors that are most strongly related to physical activity among individuals with schizophrenia. For example, the Transtheoretical Model (9, 10), Self-Determination Theory (11), and Protection Motivation Theory (12) appear to be applicable frameworks to understand and predict physical activity within this population. More recently, Twyford and Lusher (13) tested the applicability of the Theory of Planned Behavior in predicting exercise intention and behavior among 105 individuals with schizophrenia and 109 community controls. Self-efficacy, perceived behavioral control, and health professional support predicted 33.4% of variance in intention to exercise. Fruit and vegetable intake and self-efficacy accounted for 12.7% of the variance in exercise behavior.

One gap in these theoretically informed studies is that they have all been cross-sectional in design. It is not known, for example, if increasing self-efficacy causes an increase in physical activity in this population. Identifying causal relationships is necessary for identifying the most salient determinants to target in future physical activity interventions (14). Given the lack of prospective theory-based research in this population, the relative importance of potential modifiable physical activity determinants specific to persons with schizophrenia still needs to be identified (15).

To address this concern, we prospectively examined the theoretical determinants of moderate to vigorous physical activity (MVPA) in the schizophrenia population. We used the Health Action Process Approach (HAPA) (16, 17) model to undertake this research as it allowed for the examination of different social–cognitive predictors across two phases of behavior change—motivation and volition (18). During the preintentional motivation phase, beliefs related to risk perceptions, outcome expectancies, and confidence to perform the target behavior (task self-efficacy) are predictive of one’s intention to enact the behavior. Meanwhile, in the second, postintentional volition phase, self-regulatory beliefs pertaining to planning, initiating, and maintaining the behavior (e.g., action and coping planning and maintenance self-efficacy) are most salient to behavior change. This two-phased approach of the HAPA is very fitting to the schizophrenia population where motivation impairments are a common negative symptom of the illness (19).

The utility of HAPA for developing tailored health behavior interventions in various populations suggests that it would be an appropriate theoretical framework for promoting physical activity in the schizophrenia population. Within the first stage of our research, we adapted and piloted a HAPA-based inventory to capture the salient, modifiable determinants of MVPA. After initial development with participant input, the inventory was assessed for internal consistency and test–retest reliability with 25 outpatients. Preliminary support was found for the criterion validity of the inventory, adequate to excellent indices for consistency, and reliability for all but two scales (20). In this second stage, we prospectively examine the utility of the constructs within the HAPA model for predicting objectively measured MVPA in a larger sample of adults with schizophrenia. Consistent with the underlying assumptions of the HAPA model (16, 17), we hypothesized that, once controlling for key demographic characteristics, (a) risk perceptions, outcome expectancies, and task self-efficacy at time 1 would be significant predictors of intentions at time 1, (b) intentions, action and coping planning, and maintenance self-efficacy at time 2 would be significant predictors of MVPA behavior at time 3, and (c) social support would be a significant predictor of both MVPA intentions and behavior.

## Materials and Methods

### Study Design

A prospective, 4-week cohort design with three measurement points [baseline (T1), week 2 (T2), and week 4 (T3)] was used to examine the HAPA constructs and MVPA intentions and behavior across a sample of community-dwelling adults with schizophrenia. This measurement period was consistent with guidelines for testing the assumptions of the HAPA model (16, 17, 21).

### Participants

Research ethics approval was obtained from the Centre for Addiction and Mental Health (CAMH), Toronto, ON, Canada, and through the University of Toronto in September 2013. Study referral occurred through nurses, psychiatrists, and other studies at CAMH. All sessions were completed in a designated meeting room at either CAMH or the Mental Health and Physical Activity Research Centre at the University of Toronto. Similar to the first phase (20), all participants were required to have a diagnosis of schizophrenia or schizoaffective disorder, be between the ages of 18 and 64 years (22), and outpatients or inpatients with full privileges. Participants were screened on the phone by a nurse and excluded if they had been hospitalized within the past 12 months for angina pectoris, myocardial infarction, congestive heart failure, or cardiac surgery of any kind, or currently had uncontrolled hypertension (i.e., blood pressure >140 systolic/90 diastolic). Diagnosis and substance dependence/abuse were confirmed after consent was obtained using the Mini-International Neuropsychiatric Interview [MINI; (23)]. Based on Green’s (24) sample size guidelines for a regression analysis with nine predictor variables, a sample of 113 participants was necessary to detect medium-sized effects at α = 0.05 and power of 0.80. This sample size would allow for a 30% attrition rate based on earlier clinical and research experiences with this population (25, 26) and is consistent with attrition rates published in earlier prospective research using the HAPA model to predict physical activity in other rehabilitation outpatients (27).

### Measures

#### Participant Psychopathology and Demographic Characteristics

A series of instruments were self-administered to obtain demographic, health, and psychopathology characteristics of the sample. Participants were required to self-report age, gender, height, weight, living arrangements, employment and marital status, educational attainment, smoking habits, and current prescribed medications. Waist circumference was measured at the umbilicus, while past MVPA behavior was assessed using the short-form version of the International Physical Activity Questionnaire [IPAQ; (28)]. The IPAQ has previously been validated in adults with schizophrenia (26).

Symptom severity was assessed using the Brief Psychiatric Rating Scale [BPRS; (29)], and the severity scale of the Clinical Global Impression [CGI-S; (30)], with higher scores representing greater mental illness severity. The anchored Apathy Evaluation Scale [AES; 18-items; (31)] was used to assess amotivation, with higher scores representing greater apathy. Internal consistency for the AES was 0.85.

#### HAPA Inventory

Seven of the 11 subscales within the original HAPA inventory for adults with schizophrenia (20) were included in the current study. These subscales included: risk perceptions, affective attitudes, task self-efficacy, MVPA intentions, action planning, coping planning, and maintenance self-efficacy. Each item was rated on a seven-point adjectival scale with anchors varying according to the content of the scales. Higher scores on each subscale represent more positive responses. All scales targeted performing at least 150 min of physical activity of at least moderate-intensity over the next week as the outcome, which is consistent with the Canadian physical activity guidelines for adults (22). Internal consistency for the subscales in the current study ranged from 0.72 to 0.95. A more detailed description of the items within each of the seven subscales is reported by Arbour-Nicitopoulos et al. (20).

#### Social Support

Social support was assessed using the 12-item Multidimensional Scale of Perceived Social Support (MSPSS) (32), which captures the perceived emotional and informational aspects of social support from family, friends, and significant others. An example of one item included within this scale is, “*I get the emotional help and support I need from my family*.” The MSPSS has demonstrated exemplary internal consistency (α > 0.89 for all three subscales), acceptable convergent validity, and strong factor loadings (>0.70) in psychiatric outpatients (33).

#### Physical Activity Behavior

MVPA behavior was assessed using 7-day accelerometry (Actigraph^®^ GT3X). This measurement period is based on best practices for accelerometer use (34), which suggest that a 7-day protocol provides a reliable and valid estimate of physical activity behavior (ICC > 75%). A 1-min epoch was used to record activity counts (35), with each participant instructed to wear their accelerometer at all times and remove the device only for water-based activities. Activity counts were converted to weekly minutes of MVPA, using Troiano et al.’s (35) established adult cut-points for physical activity, with only bouts ≥10 min being included as per the physical activity guidelines (22). A dichotomized MVPA behavior (meeting/not meeting the guidelines for adults of 150 min of MVPA per week) was then created and used as the outcome variable in the analyses.

### Procedures

Participants took part in three, in-person assessments over a 4-week time period. During the first session (T1), participants provided written consent and were administered the MacArthur Competence Assessment Tool for Clinical Research [MacCAT-CR; (36)] by a trained research assistant to assess their competence to consent. Once capacity to consent was verified, participants completed the psychopathology (i.e., MINI, AES, and CGI), health and demographic (e.g., age, weight, and waist circumference) instruments, followed by the motivational subscales of the HAPA inventory (i.e., risk perceptions, affective attitudes, task self-efficacy, and intentions), and social support measure. At the second assessment (T2), the volitional subscales of the HAPA inventory (i.e., action and coping planning, maintenance self-efficacy) were self-administered. Participants then met with the research assistant between T2 and T3 to obtain their accelerometer and detailed instructions on how to wear the device over the 7-day period. During the T3 assessment (held on week 4), participants returned their accelerometers.

#### Data Analysis

All statistical analyses were conducted using IBM’s SPSS 23.0 and, for the accelerometer data, Actigraph’s ActiLife^®^ software version 5.0. Data were first examined for missing values, outliers, and errors. Values ≥3 SDs from the mean were removed as outliers (37). For the accelerometer data, participants had to have ≥10 h of wear time for at least four of the seven days to be included in the analyses (34, 35). Mean wear time on each of the valid days was 14.1 (1.9) h.

Descriptive analyses were conducted to examine demographic, health, and psychopathology characteristics and scale means and SDs. Hierarchical regression analyses were then used to test the ability of the HAPA model to predict MVPA intentions and behavior, controlling for age, gender, BMI, and previous self-reported MVPA behavior. In accordance with the HAPA model (16, 17, 21), in the first linear regression, intentions at T1 were regressed onto the motivational HAPA constructs at T1 (risk perception, affective attitudes, and task self-efficacy) and social support. In the second logistic regression analysis, MVPA behavior (meeting vs. not meeting the physical activity guidelines) assessed via accelerometry at T3 was regressed onto T1 social support and intentions followed by T2 action and coping planning, and maintenance self-efficacy.

## Results

### Participant Characteristics

A total of 141 participants were screened for study eligibility, 9 of whom were deemed ineligible as a result of not having the capacity to provide consent (*n* = 2) or not having a diagnosis of schizophrenia (*n* = 7). Of the remaining 132 eligible participants, 101 (76.5%) completed all three assessments as well as having valid accelerometer data. Table 1 summarizes the sample characteristics of these 101 participants. Overall, the sample was judged by the research team to be generally representative of the larger outpatient schizophrenia population at CAMH, exhibiting similar symptom severity and apathy scores, and high rates (52%) of obesity.

**Table 1:** Summary of participant demographics (*N* = 101).

Demographics	Value
Gender (male:female)	60:41
Age, mean (SD) (years)	41.5 (11.7)
**Diagnosis**	
Schizophrenia	68
Schizoaffective	32
Psychosis NOS	1
**Symptom severity**	
BPRS mean score (SD)	33.5 (7.3)
CGI mean score (SD) (*n* = 98)	3.4 (1.1)
AES mean score (SD) (*n* = 99)	31.3 (7.9)
**Medication**	
Combination therapy	18
Clozapine	18
Olanzapine	15
Risperidone	12
Aripiprazole	10
Quetiapine	7
Paliperidone	5
Loxapine	3
Ziprasidone	3
Flupenthixol	2
Zuclopenthixol	2
Haloperidol	1
Fluphenazine	1
Perphenazine	1
Did not report/unsure	3
CPZ equivalents, mean (SD) (mg)	781.4 (1,277.2)
**BMI (*n* = 100)**	
Mean (SD)	31.2 (7.8)
Normal weight (BMI < 25)	19
Overweight	29
Obese (BMI > 30)	52
**Waist circumference (*n* = 97) (cm)**	
Mean (SD)	101.7 (24.5)
Male, mean (SD) (cm)	99.8 (24.3)
Female, mean (SD) (cm)	104.5 (24.7)
IDF central obesity	73
**Physical activity levels**	
Weekly MVPA minutes,^a^ Mean (SD) (*n* = 99)	214.6 (228.9)
Meeting the physical activity guidelines^b^	28
**Living arrangement**	
Independent	51
Group home	18
With family	32
**Ethnicity**	
White	58
African	19
South Asian	6
Asian	7
Other	11
**Employment (*n* = 100)**	
Not employed	59
Student	3
Part-time	28
Full-time	2
Retired	2
Other	6
**Education (*n* = 100)**	
Some high school (no diploma)	17
High school diploma	24
Postsecondary education	58
Other	1
**Marital status**	
Single	88
Married	7
Separated	1
Divorced	5
**Smoking habits**	
Current smokers	49
Mean (SD) cigarettes/day	7.3 (10.5)
Mode cigarettes/day	10

*All values are counts unless otherwise specified. IDF (38) value represents the number of participants meeting or exceeding the cut-point for central obesity by gender and ethnicity*.

*^a^Assessed through self-report (IPAQ)*.

*^b^Assessed through 7-day accelerometry*.

*CPZ, chlorpromazine equivalents (39); IDF, International Diabetes Federation*.

### Motivational HAPA Constructs Predicting MVPA Intentions

Table 2 summarizes the mean motivational HAPA subscale scores, along with the regression coefficients, significance tests, effect sizes, and variance explained in intentions for engaging in MVPA behavior. Overall, the model explained 46% (*R*^2^_adjusted_ = 0.42, SE = 1.38) of the variance in MVPA intentions, with the motivational HAPA constructs accounting for 28% of the total explained variance. Affective attitudes (β = 0.33, *p* < 0.01) and task self-efficacy (β = 0.25, *p* < 0.05) were the only significant predictors of intentions.

**Table 2:** Summary of hierarchical linear regression (*N* = 99) of motivational HAPA stage constructs predicting MVPA intentions.

Predictor	Mean (SD)	B (SE)	95% CI	β	ΔR^2^	*t*	*p*
**Step 1: demographics**							
Previous MVPA	214.65 (288.87)	0.001 (0.001)	0, 0.002	0.15	**0.17**	1.68	<0.10
Gender	1.41 (0.50)	0.21 (0.31)	−0.40, 0.82	0.06		0.69	0.49
Age	41.45 (11.65)	0.02 (0.01)	−0.004, 0.05	0.13		1.64	0.10
BMI	31.21 (7.85)	0.004 (0.02)	−0.04, 0.04	0.02		0.18	0.86
**Step 2: motivational HAPA constructs**							
Risk perceptions	3.06 (1.49)	0.13 (0.10)	−0.07, 0.33	0.11	0.28*	1.34	0.18
Affective attitudes	5.05 (1.42)	0.42 (0.13)	0.16, 0.69	0.33**		3.15	0.002**
Task self-efficacy	5.02 (1.64)	0.27 (0.11)	0.05, 0.50	0.25*		2.39	<0.02*
**Step 3: social support**	5.04 (1.24)	0.22 (0.13)	−0.03, 0.47	0.15	**0.02**	1.76	0.08

*R^2^ values represent Nagalkerke R^2^*.

*p ≤ 0.05.

**p ≤ 0.01.

### Volitional HAPA Stage Constructs Predicting MVPA Behavior

Table 3 summarizes the mean volitional HAPA subscale scores, along with the odds ratios and 95% confidence intervals, regression coefficients, significance tests, and variance explained in MVPA behavior. Overall, the model accounted for 39% of the variance in meeting the physical activity guidelines, with intentions and social support explaining 8% of the total variance, and the volitional HAPA constructs explaining an additional 3% of the total variance. No significant relationships were exhibited between the HAPA constructs or social support and MVPA behavior.

**Table 3:** Summary of hierarchical logistic regression (*N* = 99) of volitional HAPA constructs predicting MVPA behavior assessed via accelerometry at T3.

Predictor	Mean (SD)	OR, 95% CI	β (SE)	ΔR^2^	Wald statistic	*p*
**Step 1: demographics**						
Previous MVPA	214.65 (288.87)	1.00 (1.00, 1.00)	0.00 (0.001)	**0.28**	0.01	0.94
Gender	1.41 (0.50)	0.38 (0.13, 1.09)	−0.98 (0.54)		3.26	0.07
Age	41.45 (11.65)	0.92 (0.87, 0.96)	−0.09 (0.03)		11.34	0.001**
BMI	31.21 (7.84)	0.90 (0.84, 0.98)	−0.10 (0.04)		6.29	0.01**
**Step 2: intentions and social support**						
Intentions	5.02 (1.80)	1.39 (0.95, 2.04)	0.33 (0.20)	**0.08**	2.88	0.09
Social support	5.04 (1.25)	0.74 (0.47, 1.16)	−0.31 (0.23)		1.77	0.18
**Step 3: volitional HAPA constructs**						
Action planning	4.24 (1.68)	1.12 (0.69, 1.83)	0.12 (0.25)	**0.03**	0.22	0.64
Coping planning	3.79 (1.65)	0.88 (0.54, 1.43)	−0.13 (0.25)		0.26	0.61
Maintenance self-efficacy	4.49 (1.25)	1.60 (0.90, 2.82)	0.47 (0.29)		2.58	0.11

*R^2^ values represent Nagalkerke R^2^*.

**p ≤ 0.01.

Significant negative relationships were found between age and BMI and MVPA behavior, suggesting that lower BMI and greater age, respectively, were predictive of meeting the guidelines.

## Discussion

Our findings provide partial support for the utility of the HAPA model in predicting MVPA intentions, yet not MVPA behavior, among individuals with schizophrenia. The model was able to explain nearly a third of the variance in MVPA intentions, while only 3% of the variance was explained in MVPA behavior. Affective attitudes and task self-efficacy predicted intentions. No significant relationships were found for the volitional HAPA variables and MVPA behavior.

Our findings highlight distinct roles for both affective attitudes and task self-efficacy on MVPA intentions. Given the limited role of other HAPA variables in predicting intentions and behavior, the findings suggest that more parsimonious theoretical frameworks might be of value in informing intervention work in this population. Grounding physical activity interventions in the tenets of social cognitive theory (40) is one possible starting point. Self-efficacy, a situation-specific form of self-confidence, is integral to social cognitive theory and is a consistent predictor of behavior change in various situations (41). Self-efficacy has also been a consistent correlate of physical activity in this population (12, 13, 42), and our findings add further support to the importance of this construct using a prospective design. Further research is required to develop and evaluate the effectiveness of self-efficacy-based interventions that target MVPA intentions and behavior in persons with schizophrenia. In particular, studies are required that target changes in self-efficacy and include mediational analyses to determine whether changes in self-efficacy explain changes in physical activity. It should be noted, however, that such hypothetical links have not always been demonstrated among obese adults (43).

Such interventions could be informed by Bandura’s (40) sources of self-efficacy—past performance, vicarious experiences, social persuasion, and physiological/affective factors. Past performance (i.e., mastery) is commonly considered the most powerful source of an individual’s self-efficacy. Adopting a gradual approach to increasing (and ultimately maintaining) physical activity through the use of self-monitoring and goal setting is recommended. Vicarious experiences, seeing others succeed, particularly those similar to oneself, is another source of self-efficacy. Creating opportunities to observe influential others (e.g., friends, relatives, and/or individuals with schizophrenia) performing physical activity could be considered. Notably, social support was not a significant predictor of intentions or behavior in our analyses. This may be a measurement issue as the instrument used was not specific to a physical activity context. Rather, the MSPSS assesses a more general perception of support and it may be that more tangible forms of support (e.g., informational and emotional) are required to assist individuals with schizophrenia adopt as well as maintain physical activity participation (44).

Social persuasion concerns verbal and non-verbal strategies used by others to promote self-efficacy. As others have noted, long-term support to participate in physical activity by health professionals may be necessary for some individuals (45, 46). Health professional support predicted intention to exercise in the study reported by Twyford and Lusher (13). Practical facilitation in which a health professional organizes physical activity participation and possibly attends each physical activity session in person to provide verbal encouragement, reassurance, and support may be vital (45) and reflects the nature of the intervention in one of the first exercise and schizophrenia studies reported (47).

Finally, one’s physiological and affective states can also be a source of, or threat to, self-efficacy. As one example, an individual’s perception of physiological responses to exercise might alter that individual’s self-efficacy. The extent to which a bout of physical activity makes someone feel bad rather than good may be associated with non-adherence to physical activity. Affective attitudes were related to intentions for MVPA in our study. Systematic reviews have also shown a moderate relationship between affective judgment (enjoyment) and physical activity (48). Irrespective of risk perceptions and the instrumental benefits of physical activity, greater priority might be placed on ensuring individuals enjoy the experience of physical activity. A recent acute study confirms that individuals with schizophrenia can derive acute feelings of pleasure from exercise (49). Practically, this suggests recommending participants be physically active in a way that maintains a constant or improving (but not diminishing) level of pleasure (50). Individuals could be encouraged to monitor how they feel before, during, and after bouts of physical activity in order to regulate how they feel more reliably. This may alleviate feelings of low energy commonly reported by individuals with schizophrenia (51).

Notably, MVPA was not determined by intention as Twyford and Lusher (13) also reported in their study of the Theory of Planned Behavior using a self-report measure of physical activity. This is not an uncommon finding and may reflect an “intention-behavior gap.” While intention is a critical psychological determinant of physical activity within HAPA and the Theory of Planned Behavior, it is clear that the best intentions do not always lead to behavior. Planning is one post-intentional self-regulatory strategy that is theorized to assist with when, where, and how an intention will be translated into action. However, planning did not predict MVPA in our sample. Measurement may also have been an issue in this instance. Behavior was measured objectively through accelerometry while intentions and other psychosocial constructs were assessed through self-report. Given modest correlations between self-reported and objectively measured physical activity (52), there may be a subsequent lack of correspondence between the psychosocial constructs and MVPA.

This is not to suggest planning may not have a role to play in future interventions. A review of mediators of behavior change in physical activity interventions indicates that self-regulatory constructs (e.g., planning, contingency strategies, and self-monitoring) are the most consistent agents of change in otherwise healthy adults (53). A more recent review similarly found action planning to be significantly associated with positive changes in self-efficacy (43). Planning may support behavior change indirectly via improvements in self-efficacy. Further investigation of how planning can be linked to self-monitoring and goal setting in this population is warranted.

While not the primary focus of this study, age and BMI were significant predictors of MVPA behavior, albeit not intentions. Participants with lower BMI values were more likely to be meeting the physical activity guidelines as determined via accelerometry. Given the higher rates of obesity and type 2 diabetes among individuals with schizophrenia (54, 55), this finding further illustrates the importance of integrating physical activity into clinical care. Of interest, older participants in our study were more likely to meet the guidelines than younger respondents. Although reasons for this finding can only be speculated upon it may be that having lived longer with the disorder these individuals are coping more effectively in general and this is reflected in higher levels of habitual physical activity.

There are a number of strengths as well as limitations to note for this study. This is the first prospective study assessing the theoretical determinants of objectively measured physical activity among individuals diagnosed with schizophrenia or schizoaffective disorder. However, due to inadequate power, path analysis or structural equation modeling was not possible and these would have produced a more comprehensive analysis incorporating measured variables and latent constructs, and specifying measurement error. Regression analyses are recommended to test the HAPA model in such cases (56). Even with our regression models we were slightly underpowered to fully test the effects of the relationships between the HAPA constructs and MVPA. While our physical activity behavior outcome was aligned with national physical activity guidelines, i.e., achieving at least 150 min of MVPA per week (22), dichotomizing a continuous variable comes with important limitations to recognize such as loss of information and power (57, 58). However, dichotomizing continuous data is justified when the distribution of an outcome variable is highly skewed (57), which was the case for the physical activity behavior data in this study.

The first step in developing effective interventions is to identify theoretically informed and modifiable determinants of physical activity behavior change. Our findings provide one framework on which to build physical activity interventions, and measures have been developed for evaluating social-cognitive constructs potentially mediating behavior change within this clinical population. Although limited evidence was found for the utility of the HAPA model in predicting MVPA behavior, the results suggest a need for interventions targeting self-efficacy and affective attitudes with ongoing support from health professionals.

## Ethics Statement

This study was carried out in accordance with the recommendations of the Research Ethics Boards of the Centre for Addiction and Mental Health (CAMH) and the University of Toronto with written informed consent from all subjects. All subjects gave written informed consent in accordance with the Declaration of Helsinki. The protocol was approved by the Research Ethics Boards of the Centre for Addiction and Mental Health (CAMH) and the University of Toronto.

## Author Contributions

KA-N and GF designed the study. All authors contributed to data analysis and interpretation. KA-N and GF developed the first draft of the article. All authors contributed to critical revisions of the article, and gave final approval of the version to be submitted.

## Conflict of Interest Statement

The authors declare that the research was conducted in the absence of any commercial or financial relationships that could be construed as a potential conflict of interest.
